# Immunotherapy for Recurrent Glioma—From Bench to Bedside

**DOI:** 10.3390/cancers15133421

**Published:** 2023-06-30

**Authors:** Yi Pu, Guanyu Zhou, Kejia Zhao, Yaohui Chen, Shensi Shen

**Affiliations:** 1 Laboratory of Mitochondria and Metabolism, Department of Burn and Reconstructive Surgery, West China Hospital, Sichuan University, Chengdu 610041, China; 2 Institute of Thoracic Oncology, West China Hospital, Sichuan University, Chengdu 610041, China; 3 Frontiers Science Center for Disease-Related Molecular Network, West China Hospital, Sichuan University, Chengdu 610041, China; 4 Department of Thoracic Surgery, West China Hospital, Sichuan University, Chengdu 610041, China; 5 National Clinical Research Center for Geriatrics, West China Hospital, Sichuan University, Chengdu 610041, China

**Keywords:** recurrent glioma, immunotherapy, clinical trials

## Abstract

**Simple Summary:**

Glioma is the most common and aggressive brain tumor worldwide, and most patients suffer from a recurrence. Additionally, recurrent glioma is often resistant to chemotherapies and radiotherapy. Hence, immunotherapy has come into people’s sights. The most-used immunotherapy is immune checkpoint blockade (ICB), which has shown encouraging efficacy when combined with other immune strategies, especially with antiangiogenetic antibodies. Other promising immune regimes include multiple immunotherapies which function through different mechanisms, such as oncolytic viruses, chimeric antigen receptor T cell therapies and vaccination strategies. In this review, we discuss current immune therapies applied to recurrent glioma, based on the literature of preclinical animal models, and current ongoing clinical trials published in the last 5 years. These immunotherapies have been proved to be safe and tolerant, while some combinational regimes have provided satisfying efficacy on a subgroup of patients with specific gene mutation backgrounds. Though great progress has been made, further exploration of different combination strategies is needed.

**Abstract:**

Glioma is the most aggressive malignant tumor of the central nervous system, and most patients suffer from a recurrence. Unfortunately, recurrent glioma often becomes resistant to established chemotherapy and radiotherapy treatments. Immunotherapy, a rapidly developing anti-tumor therapy, has shown a potential value in treating recurrent glioma. Multiple immune strategies have been explored. The most-used ones are immune checkpoint blockade (ICB) antibodies, which are barely effective in monotherapy. However, when combined with other immunotherapy, especially with anti-angiogenesis antibodies, ICB has shown encouraging efficacy and enhanced anti-tumor immune response. Oncolytic viruses and CAR-T therapies have shown promising results in recurrent glioma through multiple mechanisms. Vaccination strategies and immune-cell-based immunotherapies are promising in some subgroups of patients, and multiple new tumor antigenic targets have been discovered. In this review, we discuss current applicable immunotherapies and related mechanisms for recurrent glioma, focusing on multiple preclinical models and clinical trials in the last 5 years. Through reviewing the current combination of immune strategies, we would like to provide substantive thoughts for further novel therapeutic regimes treating recurrent glioma.

## 1. Introduction

Glioma, with an incidence of 6 per 100,000 population worldwide, is the most common and aggressive primary tumor of the central nervous system [1]. According to the WHO 2021 classification of central nervous system tumors (WHO CNS5), gliomas can be divided into four grades according to clinical features, histological diagnosis and molecular biomarkers (including gene mutation) [2]. Grade 3 and grade 4 gliomas are defined as “high-grade” gliomas, which have a 2-year survival rate of less than 20% [3]. The leading cause of death in high-grade gliomas is tumor recurrence. More than 90% of grade 4 glioma patients experience a recurrent tumor in situ, even with the standard of care (SOC) [4]. 

The current SOC of initial glioma is maximal safe resection (for tumor volume reduction, accurate pathological diagnosis, and gene mutation detection), followed by radiotherapy and daily temozolomide (TMZ). Additional low-density tumor-treating fields (TTF) can be applied [5]. However, there is no SOC for recurrent or therapy-resistant glioma, and options are less well-defined. One of the obstacles for new drug development and delivery strategies is the brain–blood barrier (BBB), which prevents most antitumor drugs from entering the brain. The other is the complicated tumor immune microenvironment (TME), which is the main reason for glioma immune escape and recurrence.

Multiple studies have showed that glioma has an immunosuppressive nature, and the crosstalk between tumor cells and TME can lead to resistance and recurrence. On one hand, glioma cells express higher level of programmed cell death 1 ligand (PD-L1) and indolamine 2,3-dioxygenase (IDO), which limits the presentation of antigens [6]. On the other hand, glioma has an immunosuppressive TME, leading to less-effective tumor killing. Tumor-infiltrating lymphocytes and macrophages consist of the major infiltrating immune cells in TME. In glioma microenvironment, M2 macrophage detection frequency is related to rapid tumor recurrence after radiotherapy [7]. At tumor sites, exhausted phenotypes of CD4+ and CD8+ T cells (defined as PD1+, TIM3+, LAG3+ T cells) are higher than those detected in matched peripheral blood mononuclear cells [8]. Thus, a comprehensive understanding of the nature of glioma-suppressive immunity and the tumor microenvironment can help with better immune therapy strategies.

Since the discovery of negative immune checkpoint regulator inhibition, immune checkpoint blockade (ICB) has been in the leading position for immune therapy for cancer treatment. However, the therapeutic effect of a single ICB regime for recurrent glioma remains controversial [9]. Many possible therapeutic targets, such as vascular endothelial growth factor (VEGF) and IL-13 receptor α2 (IL-13Rα2), have been discovered, as the mechanistic research probes deeper to elucidate the nature of glioma. In addition, the discovery of novel therapeutics such as oncolytic viruses (OVs) and chimeric antigen receptor T (CAR-T) cell therapies have brought enlightenment as to recurrent gliomas. 

In this review, we concentrated on articles describing current immunotherapies for recurrent glioma. Focusing on literatures of multiple preclinical animal models and clinical trials in the last 5 years, we extended our literature search to some earlier articles on therapy mechanisms that may help make the context easier to understand. The aim of this review is to elucidate different immune strategies for recurrent glioma and to discuss the possibility of further studies for novel therapeutic regimes.

## 2. Recurrent Glioma Features

Recurrent glioma often refers to grade 3 and grade 4 gliomas consisting of astroglioma and glioblastoma (GBM). According to the fifth edition of the WHO classification of tumors of the central nervous system [2] (published in 2021), astroglioma is IDH-mutant while GBM is IDH-wildtype. In this review, we will focus on recurrent IDH-mutant grade 3/4 astroglioma and IDH-wildtype GBM. Astroglioma is IDH-1/-2 mutated and often harbors TP53/ATRX mutation without 1p/19q codeletion. In a recent published article [10], it was reported that, in recurrent astroglioma, treatment of TMZ can induce hypermutation, leading to higher levels of proliferating stem-like neoplastic cells and deletion of cell-cycle regulators CDKN2A, as well as amplification of CCND2. Additionally, the immune cell composition also changes at recurrence. Compared with primary astroglioma, recurrent astroglioma has an obvious decreased expression of brain-resident microglia signature and increased acquisition of HLA loss of heterozygosity. In recurrent GBM, the tumor–neuron interaction causes higher leading-edge content, which is a proneural subtype and retains neural tissue characteristics [10]. The myeloid compartment in recurrent GBM shows a high blood-driven macrophage signature, which is an immunosuppressive phenotype expressing PDCDLG1 and IDO1. These features suggested that glioma had undergone changes in cell states in association with genetic and microenvironment changes through initial treatment of TMZ and radiotherapy, necessitating other kinds of treatment. 

## 3. Immune Checkpoint Blockade

Immune checkpoint blockade (ICB) is a turning point in anti-tumor treatment in many cancers. So far, more than ten immune checkpoints have been identified and proved to be promising therapy targets, such as lymphocyte activation gene-3 (LAG-3), indoleamine 2, 3-dioxygenase 1 (IDO1) and T cell immunoglobulin and mucin-domain containing-3 (TIM- 3). However, only antibodies targeting cytotoxic T lymphocyte antigen 4 (CTLA-4) and programmed cell death protein 1 (PD-1)/programmed cell death-ligand 1 (PD-L1) have been approved by FDA and widely used (Table 1).

### 3.1. CTLA-4/B7 Axis

**Figure 1:**
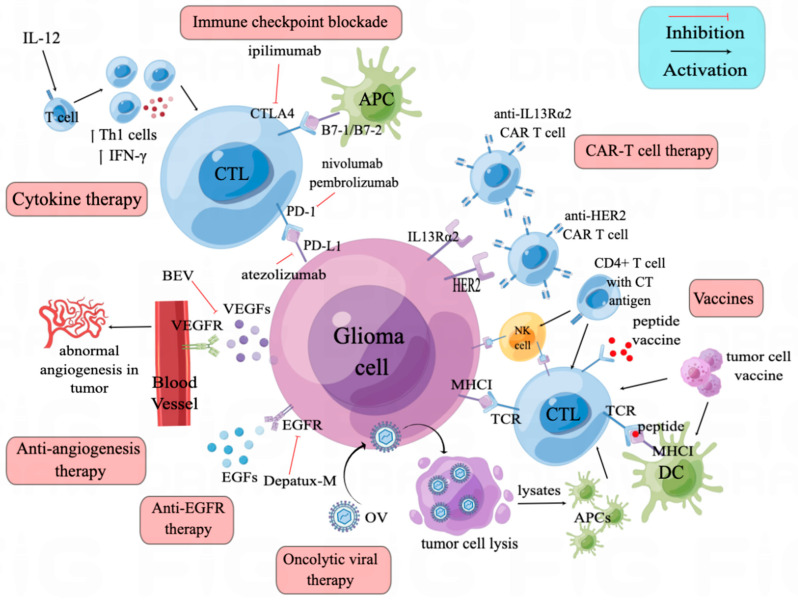
Mechanisms of current immunotherapies employed in recurrent glioma. Glioma cells and the tumor microenvironment often activate immune checkpoint ligands for PD-1 and CTLA-4 to escape immune eradication. Inhibition of PD-1 and CTLA-4 can effectively intercept the reaction. VEGF (vascular endothelial growth factor) is an angiogenesis inducer and is upregulated in glioma. Anti-VEGF antibody can prevent binding of VEGF-A and VEGF receptor tyrosine kinase (VEGFR) as well as neovascularization. About half of recurrent-glioma patients have amplification of epidermal growth factor receptor (EGFR). Anti-EGFR antibody can prevent the binding of EGF and EGFR, thus preventing tumor cell proliferation. IL-12 is an anti-tumor cytokine that can stimulate shifting of CD4+ T cells to the Th1 phenotype and increase IFN-γ secretion. Oncolytic viruses (OVs) can cause glioma cell lysis directly and the released tumor cell lysates can be recognized by antigen presenting cells (APCs) to induce an anti-tumor immune response. Dendritic cells (DCs) are efficacious APCs and can activate cytotoxic T lymphocytes (CTLs) to kill glioma cells. DCs can be co-cultured with tumor cell lysates to act as a DC vaccine for glioma tumor killing. Additionally, tumor cell lysates or glioma stem cells themselves can be used as vaccines to stimulate an anti-tumor immune response. Autologous CD4+ T helper cells expressing endogenous cancer/testis (CT) antigens can also be used as vaccines to activate CTLs and NK cells. NK cells are innate immune cells which can be used directly as vaccines to lyse tumor cells. Peptides mimicking several proteins can also be utilized as vaccines to induce specific anti-tumor immune responses. Tumor-associated antigens (TAAs) such as IL13Rα2 and EGFRνIII can be recognized by CTLs by means of the promotion of MHC-1 expression. These TAAs are being exploited as targets of genetically modified CAR-T cells.

CTLA-4, expressed by T cells, is a homolog to CD28. The competitive binding of CTLA-4 with CD80 (B7-1) and CD86 (B7-2) is not associated with a proliferation of T cells, but does increase T cell survival and differentiation, while also preventing the co-stimulatory signal provided by CD28 and B7 binding [11,12] (Figure 1). Though the anti-CTLA-4 antibody, ipilimumab, has been tested and found effective in treating several cancers, preclinical glioma models have brought unsatisfying results as to anti-CTLA-4 monotherapy [11,13]. Single anti-CTLA-4 therapy has neither improved symptom-free survival in GL261 glioma mouse model, nor enhanced the costimulatory capacity of antigen-presenting cells (APCs) [13]. However, multiple preclinical studies combining the anti-CTLA-4 antibody with other therapeutics have had encouraging results. The same study using a GL261 glioma mouse model showed complete tumor regression using a sequential regime of an anti-CTLA-4 antibody and a whole-tumor-cell vaccine [13]. Other studies combining anti-PD1 with anti-CTLA-4 in GL261 glioma mouse models showed decreased tumor growth and improved symptom-free survival [14,15]. Thus, clinical trials of the FDA-approved anti-CTLA-4 antibody ipilimumab focus on combination strategies. A recently registered phase 2 randomized clinical trial (ISRCTN84434175) aimed to compare the efficacy of a combination of ipilimumab and chemo agent TMZ with TMZ monotherapy [16]. Other clinical trials of anti-CTLA-4 combining anti-PD1/anti-PD-L1 will be reviewed in Section 3.2.

### 3.2. PD-1/PD-L1 Axis

In glioma microenvironments, PD-1 is expressed mainly by T cells, while PD-L1 can be expressed by many cells, including glioma cells, microglia/macrophages and dendritic cells (DCs) [17,18] (Figure 1). Some of the immune-related cytokines, such as interferon-γ (IFN-γ), tumor necrosis factor α (TNFα) and interleukin-12 (IL-12), play a role in inducing PD-L1 expression. IFN-γ, a pro-inflammatory cytokine produced by T cells and NK cells, can bind to its receptor, interferon-gamma receptor (IFGR) and activate the Janus kinase signal transducer and activator of transcription (JAK-STAT) pathway through STAT1. The downstream expression of interferon-responsive factor 1 (IRF1) mediates IFN-γ-induced PD-L1 [19]. TNFα can also induce PD-L1 expression by activating signal molecules such as NF-κB, IκB [20]. On the other hand, IL-12 plays a dual role in PD-L1 expression. In monocyte-derived macrophages, secretion of IL-12 can upregulate PD-L1 expression, while in THP-1-derived macrophages, PD-L1 expression is downregulated by IL-12 [21]. Binding of PD-L1 to the PD-1 receptor activates the PD-1/PD-L1 axis, inhibiting T cell proliferation by activating protein tyrosine phosphatase SHP2 and dephosphorylating Zap70 [12,17]. The increased level of PD-L1 in glioma correlates to FOXP3 expression, which is a marker for the immunosuppressive regulatory T cells (Tregs) [22].

In preclinical glioma mouse models, strategies combining an anti-PD-1 antibody with other therapeutics have been proved effective. In the immunogenic GL26 tumor model, anti-PD-1 monotherapy prolonged median survival from 24 days (vehicle control) to 28.5 days. When combined with TMZ and anti-Na/H exchanger isoform 1 (anti-NHE1), median survival extended to about 41 days [23]. Other research combining anti-PD-1 with TMZ obtained similar survival rates with increased CD8+ T cell/Treg ratios [24,25]. The effect of anti-PD-L1 combination regimes is also encouraging. A study showed 60% of mutant isocitrate dehydrogenase 1 (mIDH1)-glioma-bearing mice had complete tumor regression with reduction of exhausted T cells and generation of memory CD8+ T cells after receiving the combination of anti-PD-L1 antibody, 2-hydroxyglutarate (D-2HG) inhibition, irradiation and TMZ [26]. In a TMZ-refractory glioma mouse model, the combination of anti-PD-L1 antibody and p38MAPK inhibitor significantly improved the 60-day survival rate from 0 (vehicle control) to 60%. The dissociation of post-treatment tumor and flow cytometry showed a decrease of F4/80+/CD11b+ macrophages/microglia [27]. 

Nivolumab and pembrolizumab are both FDA-approved anti-PD1 antibodies commonly used for advanced-stage melanoma. In recurrent glioma, the safety of both antibodies has been verified in several retrospective and phase 1 studies, while the efficacy of monotherapy remains controversial [28,29,30,31,32,33,34,35]. In addition to patient selection, multiple combination regimes have been studied in clinical trials for recurrent glioma. One of the most-used strategies is the combination of nivolumab and ipilimumab, an anti-CTLA4 antibody. A study analyzed the differentiation status of CD8+ tumor-infiltrating lymphocytes (TILs) in primary glioma patients to sort out the underlying mechanisms of non-responsiveness to ICB treatment [36]. The result suggested that PD1+ CD8+ T cells exhibited a more terminally differentiated phenotype (Eomes^hi^T-bet^lo^) and correlated with the response to anti-PD1 therapy. When applying anti-CTLA4 and anti-PD1 combinational therapy, patients with low Eomes^hi^T-bet^lo^ CD8+ TILs showed additional increases of CD8+ TIL proliferation. In a nonrandomized phase 1 cohort of the Checkmate 143 study (NCT02017717), nivolumab monotherapy was proved to be better-tolerated than combinations of high doses (3 mg/kg) or low doses (1 mg/kg) of ipilimumab, while the effects of therapies were comparable [37]. To solve the problem of low activity of ICB in recurrent glioma, a phase 1 study explored intracerebral administration of nivolumab and ipilimumab after re-surgery in the resection cavity. The median overall survival rate did not improve compared with intravenous administration, though intracerebral administration was safe and feasible [38]. Combination of an anti-PD1 antibody with anti-vascular endothelial growth factor (VEGF) antibody, bevacizumab (BEV), has also been evaluated in multiple clinical trials. In a randomized phase 3 study in the Checkmate 143 study (NCT02017717), 369 first-recurrent-glioma patients were randomly assigned to nivolumab monotherapy or BEV monotherapy. While the 6-month progression-free survival (PFS-6) and overall survival (OS) rates were comparable between groups, the objective response rate was higher in BEV-treated patients (23.1% vs. 7.8%) [39]. Another phase 2 study compared the efficacy of pembrolizumab alone or in combination with BEV. Though both regimes were well tolerated, limited benefits were brought to patients with recurrent glioma [34]. Other combinations with, for example, axitinib (tyrosine kinase inhibitor of VEGF receptors), cyclophosphamide, and hypofractionated stereotactic irradiation (HFSRT), have been proved safe, but of little benefit [40,41,42]. Clinical trials of anti-PD-L1 antibodies such as atezolizumab (NCT01375842) and durvalumab have proved the safety but ineffectiveness of monotherapy or combination strategies with BEV [9,43]. 

Although the above combination strategies in recurrent glioma were not satisfying, posttreatment analysis revealed therapeutic benefit correlates to indexes such as PD-L1 expression level, baseline steroid use, peripheral lymphocyte counts and gene expression profile, suggesting novel combination regimes and patient selection [9,35].

## 4. Anti-Angiogenesis Therapy

VEGF has been known as an angiogenesis inducer and is upregulated in glioma; the binding of VEGF-A and VEGF receptor tyrosine kinase (VEGFR) can activate the VEGF signaling pathway to promote neovascularization [44,45] (Figure 1). Since the first report of an anti-VEGF antibody decreasing tumor volume in multiple cancer preclinical models in 1993, the effects of an anti-VEGF antibody, bevacizumab, have been investigated in several clinical trials [44].

Bevacizumab (BEV) is the first humanized monoclonal antibody that prevents the interaction of VEGF-A and VEGFR by binding to circulating VEGF-A [46]. After the encouraging results of the pivotal study AVF3708g in relapsed glioblastoma, BEV was approved to treat recurrent and progressing gliomas. Multiple clinical studies have explored multiple regimes in recurrent glioma. In a prospective study of 29 recurrent-glioma patients receiving only BEV (10 mg/kg, i.v., every 2 weeks till progression), the baseline level of neutrophils below 3.9 G/L and Treg above 0.011 G/L were related to prolonged OS [47]. Adding BEV (10 mg/kg, i.v.) on days 1 and 15 in the 28-day cycle of TMZ (100 mg/m^2^) improved PFS-6 up to 52%, suggesting that BEV plus bi-weekly TMZ may be a possible regime [48]. However, two other phase 2 studies (TAMIGA and TAVAREC) did not draw such promising results. In the TAMIGA trial (NCT01860638), 123 newly diagnosed glioma patients were randomized into lomustine (CCNU) plus BEV or plus placebo groups at first disease progression. However, no survival benefit was observed (median survival was 6.4 vs. 5.5 months, respectively) [49]. In addition, a combination of TMZ and BEV did not improve overall survival compared to TMZ single agent in the multicenter phase 2 TAVAREC trial (NCT01164189) [50]. Further analysis of 122 samples collected in the TAVAREC trial revealed the predictive value of homozygous deletions in CDKN2A/B for survival benefit [51]. Other than the unsatisfying clinical trials mentioned above, clinical trials combining BEV with signaling-pathway inhibitors such as Src inhibitor, VOR inhibitor, PI3K inhibitor, etc., drew similar, discouraging results [40,52,53,54,55,56,57,58]. 

## 5. Anti-EGFR Therapy

Amplification of epidermal growth factor receptor (EGFR) was observed in about 50% of glioma patients [59] (Figure 1), making anti-EGFR therapy a possible choice for glioma patients. Unfortunately, a phase 3, randomized, double-blinded clinical trial failed to show a survival benefit of the EGFR deletion mutation vaccine rindopepimut (CDX-110) on newly diagnosed EGFRνIII positive glioma patients [60]. Rindopepimut is an EGFRvIII-specific peptide conjugated to keyhole limpet haemocyanin. After enrollment, patients were randomly assigned to two groups, rindopepimut or control (keyhole limpet haemocyanin), concurrent with standard oral TMZ. At final analysis, the mOS were 20.1 months for the rindopepimut group and 20.0 months for the control group. Though around 50% patients harbored the EGFRνIII deletion, and EGFR amplification was maintained at the time of recurrence [61,62], neither EGFR inhibitors nor monoclonal antibodies targeting extracellular EGFR showed a survival benefit among recurrent-glioma patients. Depatuxizumab mafodotin (Depatux-M) is a modified antibody–drug conjugate composed of an EGFR monoclonal antibody (depatuxizumab) and a microtubule inhibitor monomethyl auristatin F (mafodotin). 

In preclinical mouse U87MG and U87MG EGFRνIII models, the combination of depatux-M, TMZ and radiotherapy inhibited tumor growth more than depatux-M alone or TMZ plus radiotherapy [63]. Additionally, the preclinical model also verified that depatux-M worked efficaciously in recurrent gliomas [63]. In a phase 1 study (NCT01800695), 1.25 mg/kg intravenous depatux-M every two weeks was well tolerated in 66 EGFR-amplified recurrent-glioma patients [64]. In another phase 1 study comparing depatux-M plus TMZ with depatux-M monotherapy in recurrent-glioma patients (NCT01800695), PFS6 was higher in the monotherapy group than in the combination group (40% vs. 26.7%, respectively), while OS was still higher in the combination group (17.9 months vs. 7.2 months). This might be due to the high EGFR amplification and mutation rate in the monotherapy group (8/9 vs. 9/15 in combination group) [65,66]. In the further multicenter phase 2 study (NCT02343406), long-term analysis of 199 events showed that a combination of depatux-M and TMZ worked effectively compared with the control group (hazard ratio 0.66), while single depatux-M did not show efficacy compared with the control group (hazard ratio 0.96) [67]. Another analysis of health-related quality of life (HRQoL) showed depatux-M had no impact on HRQoL in EGFR-amplified recurrent-glioma patients [68]. Common adverse events (AEs) among all clinical trials were ocular problems, including blurred vision, dry eyes and photophobia, most of which are grade 3/4 AEs [64,65,66,67,68,69].

## 6. Cytokine Therapy

Cytokines are secreted by immune cells and can regulate the immune response against tumors. One of the most used cytokines is interleukin, of which IL-12 showed promising anti-tumor efficacy (Figure 1). IL-12 functions mainly by increasing IFN-γ secretion and shifting CD4+ T cells to a Th1 phenotype [70]. In an advanced-glioma mouse model, combination of intratumor IL-12 with anti-CTLA-4 successfully led to tumor eradication, compared with either of the monotherapies [71]. To solve the systemic inflammatory toxicity of IL-12, locoregional injection and a regulatable “turn-on” switch were developed. The regulatable hIL-12 vector (Ad-RTS-hIL-12) was injected in the cranial cavity after surgery (NCT02026271). Patients were given different doses (from 10 mg to 40 mg) of veledimex (VDX), the oral activator of the IL-12 vector. A quantum of 20 mg VDX showed superior drug compliance, with a mOS of 12.7 months [72]. Adverse events, such as systemic inflammation, were VDX dose-related and were reversed upon VDX discontinuation [72,73].

## 7. Oncolytic Viruses

Oncolytic viruses are genetically modified viruses that preferentially infect and kill cancer cells. Upon oncolysis, new infectious oncolytic viruses will be released to infect and kill the remaining cancer cells. The most-used viral vectors include retrovirus, adenovirus and human simplex virus type-1 (HSV-1) (Figure 1, Table 2).

### 7.1. Retrovirus 

Different from HSV and adenovirus vectors, modified retroviral replicating vectors (RRVs) can specifically infect tumor cells without direct oncolytic effects on them. Thus, this makes RRVs a good platform for tumor-targeting gene therapies. In the past few years, the most used RRVs in glioma therapy is the Toca 511, which is modified to encode a transgene for yeast cytosine deaminase (yCD2). When the virus is administered into the resection cavity after surgery, it can specifically infect the remaining cancer cells; then, yCD2 can convert the oral prodrug 5-fluorocytosine (5-FC; Toca FC) into the cytotoxic 5-fluorouracil (5-FU) and kill cancer cells [74]. In a phase 1 trial (NCT01470794), median survival reached 11.9 months for all 53 patients [75]. Other studies further analyzed tumors and peripheral blood samples in this and other phase 1 trials [74,76]. First and foremost, Toca 511 is detected mainly in tumor samples and only transiently detected in the peripheral blood of some patients. Having more activated memory CD4+ T cells, more M1 macrophages with fewer resting NK cells and M0 macrophages in the tumor microenvironment is related to the better response to Toca 511. Responders also showed posttreatment elevation of E-selectin and MIP-1-beta in peripheral blood. However, in a later multicenter, randomized phase 2/3 clinical trial (NCT02414165), Toca 511 showed no advantages compared with SOC [77]. In this trial, patients were given either Toca 511/FC or SOC (investigator’s choice of single agent therapy, such as lomustine; TMZ; or BEV). The median OS was 11.1 and 12.2 months for Toca 511/FC groups and SOC groups, respectively, while the second endpoints and AE rates had no difference between groups. 

### 7.2. Adenovirus

Oncolytic adenovirus can not only directly oncolysate tumor cells but also induce antitumor immune responses [78,79]. Adenovirus VB-111 (ofranergene obadenovec) is the most studied oncolytic adenovirus for recurrent glioma. It is composed of a non-replicating adenoviral vector with a human Fas-chimera transgene, which is controlled by a modified murine pre-endothelin promoter (PPE-1-3x) [78]. VB-111 is promising on recurrent tumors for its function to disrupt neovascularization independently of the pro-angiogenic signaling pathway and to induce infiltrating CD4+ T and CD8+ T cells [78]. Even though VB-111 has been proved well-tolerated in clinical trials, the efficacy remains controversial. In a phase 1/2 study, the median survival has been significantly improved to 414 days for patients in VB-111 and BEV-combination groups [78]. The same team later conducted a phase 3 study (NCT02511405) and drew a different result. It seemed that only those patients who had both smaller tumors and a posttreatment febrile reaction had improved survival over the combination group [80]. Another adenovirus-based oncolytic virus, DNX-2401 (Delta-24-RGD; tasadenoturev) has also been proved effective and tolerated in a phase 1 study [79]. Posttreatment tumor samples showed infiltrating CD8+ T cells and T-bet+ cells, along with decreasing transmembrane immunoglobulin mucin-3, indicating an anti-tumor immune microenvironment [79]. Agaltimagene besadenovec (AdV-tk) is a modified oncolytic adenoviral vector expressing the herpes simplex virus (HSV) thymidine kinase (tk) gene. The development of AdV-tk was intended to implement a gene-mediated cytotoxic immunotherapy (GMCI) anti-tumor strategy [81]. After local delivery of AdV-tk, the anti-herpetic prodrug was given to activate the STING (stimulator of interferon genes) pathway, turning a “cold tumor” into an immune “hot” tumor. GMCI using AdV-tk has been proved to be safe and tolerated in both adult and childhood recurrent glioma with an efficacy to be further elucidated [81,82].

### 7.3. HSV-1

HSV-1 is one of the most-studied oncolytic viral vectors, as it can infect most cell types and needs a low multiplicity of infection for total cell killing. Furthermore, HSV-1 has a large genome which can be inserted by large or multiple transgenes [83]. The first oncolytic HSV-1 used in clinical trial was G207, which has deletions in both copies of the γ34.5 gene and a lacZ insertion inactivating the ICP6 gene [84,85]. In a neuroblastoma syngeneic mouse model, G207 showed both direct oncolytic activity and induction of antitumor immunity by means of increasing cytotoxic T cell activity [86]. Recent research analyzed biopsies pre- and post-G207 treatment from six recurrent-glioma patients [87]. RNA-seq analysis revealed that genes enriched in intrinsic IFN-mediated antiviral and adaptive immune functional responses correlated with survival duration. However, further clinical trials in glioma showed an unsatisfying efficacy of G207. Subsequently, G47Δ was made by deleting the α47 gene in the G207 genome [88]. This modification caused further attenuation of virus levels in normal cells and stimulated an enhanced antitumor immunity. Two clinical trials conducted by the same team showed the promising results of G47Δ in adult recurrent glioma in the Japanese population [83,89]. In the phase 1/2 study (UMIN-CTR Clinical Trial Registry UMIN000002661), G47Δ was proved safe and tolerated, with a median overall survival rate of 7.3 months [83]. Further, in another phase 2 study (UMIN-CTR Clinical Trial Registry UMIN000015995), posttreatment biopsies revealed increasing tumor-infiltrating CD4+/CD8+ lymphocytes and persistently low numbers of Foxp3+ cells [89].

### 7.4. Parvovirus

Other than above mentioned oncolytic viruses, rat parvovirus has also been applied in clinical trials of recurrent glioma. In an 18-patient phase 1/2a study of recurrent glioma, rat H-1 parvovirus (H-1PV) was safe and tolerated, with PFS6 at 27% [90]. H-1PV crossed BBB, activated macrophages and infiltrated cytotoxic T cells were detected in infected tumor samples [90].

## 8. Vaccines and Cell-Based Immunotherapies

Vaccines and cell-based immunotherapies are based on the notion of a tumor-specific immune response towards the injected exogeneous antigens. Currently, cell vaccines, cell-based immunotherapies and peptide vaccines have been applied in clinical trials of recurrent glioma (Figure 1, Table 3).

### 8.1. Immune Cell and Cancer Cell Vaccines/Cell-Based Immunotherapies

#### 8.1.1. Dendritic Cell Vaccines

Dendritic cells derive from bone marrow and are morphologically and functionally heterogenetic cells which can present antigens to CD4+ and CD8+ T cells. As the largest population of APCs, DC vaccines were the first immune cell vaccine to be studied for cancer immunotherapy. Though they are difficult to obtain, the efficacy of autologous DCs were tested in several clinical trials. In the HGG-2006 phase I/II trial, the efficacy of DC vaccine on newly diagnosed GBM was evaluated [91]. A total of seventy-seven patients were given four weekly vaccinations after the 6-week chemoradiotherapy, as well as four boost vaccinations during maintenance chemotherapy. This regime is feasible without major AEs, and the PFS6 was 70.1%. This possibly beneficial result led to further clinical trials of DC vaccines on both newly diagnosed and recurrent gliomas. In a double-blind, placebo-controlled phase 2 study, autologous DC vaccine loaded with glioblastoma-stem-cell-like lines (GSC) prolonged OS in the IDH1-wildtype TERT-mutant and B7-H4 low expression newly-diagnosed GBM patients, with increasing levels of plasma CCL22 and IFN-γ [92]. In another clinical trial, 10 recurrent-glioma patients were given a fusion of autologous DC vaccine and glioma cells after TMZ resistance [93]. The median PFS was 10.3 months and a specific immune response against chemoresistance-associated peptides (CAP), such as WT-1, gp-100 and MAGE-A3, was detected. Another study further tested the safety and efficacy of DC vaccine pulsed with lysates from a glioblastoma (GBM)-stem-cell-like cell line [94]. For the 25 recurrent-glioma patients, PFS6 was 24%, which proved the combination safe and tolerated. These clinical trials indicated that DC vaccine is safe and tolerated. Subsequently, other clinical trials have tested the possibility of a chemotherapy and DC vaccine combination [95,96,97]. In one study [95], recurrent-glioma patients were implanted with Gliadel Wafers, composed of biodegradable carmustin, after resection, followed by autologous DC vaccine pulsed with tumor cell lysates. Median PFS was 3.6 months from the beginning of vaccine therapy. In another clinical trial (HGG-2010) [96], similar DC vaccine was given before and during maintenance or after TMZ chemotherapy. There was no difference of OS between groups, and the median OS was 19 months, with increasing CTL CD69+ cells and decreasing Tregs correlating to better OS. However, in another phase 3 clinical trial (NCT00045968), autologous tumor lysate-loaded DC vaccine (DCVax-L) plus TMZ was applied in 64 recurrent-glioma patients [97]. The median OS from relapse was 13.2 months vs. 7.8 months for the control patients (those who only received SOC TMZ). These combinations were safe, however, it seemed the efficacy of DC vaccine plus chemotherapy remained controversial, compared with that of DC vaccine therapy alone. As mentioned above, autologous DC obtained from patients’ peripheral blood is difficult to prepare. Hence, allogeneic DC vaccines were proposed as an alternative regime. Several studies have demonstrated that cytomegalovirus (CMV) proteins are expressed in more than 90% of glioblastoma [98,99]. Thus, John H. Sampson and his colleagues developed a DC vaccine targeting CMV protein pp65 and conducted three clinical trials (NCT00639639) in newly diagnosed glioma [100]. In the first blinded, randomized phase II clinical trial, nearly one third of patients were without tumor recurrence at 5 years from diagnosis. In the second clinical trial, survival rate was 36% at 5 years from diagnosis. In the third study, which was the first two-arm trial, migration of the DC vaccine to draining lymph nodes was observed, and this phenomenon was recapitulated in a larger confirmatory clinical study (NCT02366728) conducted by the same team.

#### 8.1.2. Glioma (Stem) Cell Vaccines

An interesting phenomenon observed in malignant tumor patients is that patients with autoimmune diseases may have a better prognosis. This brought into a question whether a mimicry “autoimmune” state may help to eradicating cancer cells. In a syngeneic rat glioma model, allogeneic GBMs were proved to be effective for established tumors [101]. This was the first preclinical proof of ERC1671, a vaccine containing autologous and allogeneic (from other patients) GBM tumor cells and lysates. Later in a double-blinded, randomized phase 2 clinical trial of recurrent glioma [102], ERC1671 was administrated with cyclophosphamide and granulocyte–macrophage colony-stimulating factor (GM-CSF) plus BEV. Compared with those in the control group (placebo plus BEV), patients in ERC1671 obtained a 4.5-month longer OS (7.5 months vs. 12 months), with a positive correlation of maximal CD4+ T lymphocytes. In another two-arm study of low-grade glioma, the safety and effectiveness of an allogeneic cell-lysate-based vaccine GBM6-AD, a glioma stem cell line isolated from a glioma patient, was tested [103]. In this study, patients were divided randomly into two arms; the first arm received vaccine before surgery, while the second arm did not. Both arms of patients received adjuvant vaccine after surgery. The GBM6-AD was co-administrated with TLR3 ligand, polyinosinic-poly-cytidylic acid (poly-IC) stabilized with poly-lysine and carboxymethylcellulose (poly-ICLC). This coadministration helped the vaccine’s trafficking to the central nervous system. The median PFS was 11 months for all patients, while upregulation of type-1 cytokines and chemokines and increase of CD8+ T cells in peripheral blood were only found in patients in the neoadjuvant arm. In addition, neoadjuvant vaccination led to effector phenotype CD8+ T cell clones and migration to TME. 

#### 8.1.3. T-Cell-Based Immunotherapy 

One major category of adoptive immune eradication of cancer cells is the reorganization of tumor-associated antigens by cytotoxic T lymphocytes (CTL). Cancer/testis (CT) antigens, a group of >100 proteins of different families with unknown functions, are one of the major classes of heterogenous antigens recognized by CTL [104]. In cancer cells, CT antigens were usually epigenetically depressed by DNA demethylation. Hence, Walter and his colleagues [104] developed an autologous CD4+ T helper cells expressing endogenous CT antigens by treated with DNA demethylating agent. These CD4+ T helper cells can act as APCs to generate CTLs and natural killing (NK) cells in vivo. In the following 25-patient phase 1 trial, 10 of 25 patients who received all three rounds of treatment survived at the 20-week evaluation; among these, three had tumor regression.

#### 8.1.4. NK-Cell-Based Immunotherapy

The notion of using NK cells to treat malignant tumors is not a vaccination strategy. NK cells are innate immune cells which lyse stressed cells and tumor cells without a need to be previously stimulated [105]. Additionally, tumor cells often downregulate self-markers such as major histocompatibility complex (MHC) class I to escape from T cell cytotoxicity. This brings about another advantage of NK cell immunotherapy, as it makes tumor cells more susceptible to NK cell lysis [105]. In a clinical trial conducted in 2004 [106], autologous NK cells co-cultured with an irradiated human feeder cell line (HFWT) using RHAM-alpha medium supplemented with 5% autologous plasma and IL-2 were injected in nine adult recurrent-glioma patients. Clinical evaluation revealed three cases of partial response (PR), two of minor response (MR), four of no change (NC) and seven of progression of disease (PD) along sixteen courses of NK cell treatment. In another clinical trial, the safety of intraventricular infusion NK cell treatment was proved in pediatric recurrent-glioma patients [107].

### 8.2. Peptide Vaccines 

Survivin (BIRC5) is a member of a group of anti-apoptotic proteins highly expressed in glioma. SurVaxM (SVN53-67/M57-KLH) is a synthetic long peptide mimic peptide that spans amino acids 53 through 67 of the human survivin protein sequence [108]. This peptide was modified at amino acid M57 to enhance binding of the core survivin epitope to HLA-A*0201 (human leukocyte antigen) molecules. KLH, keyhole limpet hemocyanin, was used as a vaccine adjuvant. Preclinical murine glioma models had shown that SurVaxM could stimulate an anti-tumor immunity. In a safety evaluation clinical trial, nine patients with recurrent glioma that was survivin-positive, and who had either HLA-A*02 or HLA-A*03 MHC class I allele-positivity, were included. Six of eight evaluable patients developed cellular and humoral immune response against glioma, with a median PFS of 17.6 weeks. Advancement in tumor immunology has revealed that tumor-associated antigens (TAAs) can be used as cancer vaccines. Wilms’ tumor gene 1 (WT1) product is one of the TAAs that can be utilized as peptide vaccine. A phase 1 clinical trial on recurrent-glioma patients was conducted to verify the safety of a cocktail vaccine of WT1 HLA class I and II peptides [109]. Eleven of the included fourteen HLA-A*24:02-positive patients completed vaccination. The median OS and 1-year OS rate were 24.7 weeks and 36%, respectively. Another “cocktail” vaccine which has been used in clinical trials is personalized peptide vaccine (PPV), which contains four peptides chosen from forty-eight warehouse peptides according to the patient’s HLA type and preexisting peptide-specific immunoglobulin (Ig) G levels [110]. In this study, 88 recurrent-glioma patients were randomly assigned to PPV group or placebo group at a ratio of 2:1. However, this trial failed to meet both primary endpoint (OS) and second endpoint. Isocitrate dehydrogenase (IDH) mutations, a disease-defining mutation resulting in the oncogenic IDH1R132H protein, have often happened in glioma patients. A peptide vaccine targeting IDH1R132H (IDH1-vac) was proved safe and effective in stimulating immune response in a multi-center phase 1 trial (NOA-16 trial) in newly diagnosed glioma patients [111]. In a recent published paper, a randomized, three-arm, window-of-opportunity, multicenter national phase 1 trial (AMPLIFY-NEOVAC, NCT03893903) in resectable IDH1R132H-mutant recurrent-glioma patients was designed [112]. In this trial, patients will receive either IDH1-vac or avelumab (AVE), an anti-PD-L1 antibody, or both. AMPLIFY-NEOVAC is an ongoing clinical trial which aims to demonstrate the safety of this combination for enhanced IDH1-vac-induced T cells in peripheral blood.

## 9. CAR-T Therapy

Chimeric antigen receptor (CAR) T cell therapy, a special kind of T cell vaccine, has been proved effective in multiple cancers, especially in leukemia [113]. Chimeric antigen receptors on the T cell often recognize unprocessed antigens on cancer cells. In the setting of glioma, IL13Rα2, human erbb2 receptor tyrosine kinase 2 (HER2) and EGFRνIII are normally targeted proteins on the cancer cell’s surface (Figure 1, Table 3). In a pilot study, recurrent-glioma patients expressing EGFRνIII have been enrolled and given anti-EGFRνIII CAR T cells, with no clinical benefit observed [114]. Another study analyzed post-EGFRνIII CAR T cell therapy tumor samples and found antigen decrease in five of seven patients [115]. Additionally, an increase of inhibitory molecules and infiltration of Tregs were detected in the tumor microenvironment after CAR-T therapy, indicating that the local microenvironment’s adaptive change and antigen heterogeneity were related to CAR-T therapy efficacy. In a phase 1 study [116], 17 patients with HER2 positive glioma were given autologous HER2-CAR T cell infusions. Infusions were well tolerated, and median OS was 11.1 months. In another phase 1 clinical trial (NCT03500991), locoregional use of median-length HER2-CAR T cells in child or young adult recurrent-glioma patients was evaluated [117]. Interim reports showed high concentrations of CXCL10 and CCL2 in the cerebrospinal fluid, indicating local CNS immune activation. To conquer the limitation of autologous CAR T cells, an off-the-shelf, allogenic CAR T cell was made [118]. IL13Rα2-targeted CAR+ (IL13-zetakine+); cytolytic T-lymphocyte (CTL) obtained from a health donor was then genetically engineered using zinc finger nucleases (ZFNs) to permanently disrupt the glucocorticoid receptor (GR) (GRm13Z40-2) and endow resistance to glucocorticoid treatment. Six unresectable recurrent-glioma patients were recruited to evaluate the safety and feasibility of intracranial GRm13Z40-2 T cells combined with recombinant human IL2 (rhIL2, aldesleukin). The regime was to give four doses of 10^8^ GRm13Z40-2 T cells over a two-week period, along with aldesleukin (nine infusions ranging from 2500–5000 IU). This regime was well tolerated and transient tumor reduction with/or tumor necrosis at the site of T cell infusion were observed in four of six patients. In addition, the combination of humanized L13Rα2 CAR T cells and anti-CTLA4 antibody has been proved to be more effective than single agent in a glioma mouse model [119]. This was an encouraging result for further use of allogenic CAR T therapy in recurrent glioma.

## 10. Conclusions and Perspective

Though the current evidence shows no treatment benefits of immunotherapy for newly diagnosed glioma patients [120,121], great progress in translational immunotherapy for recurrent glioma has been made in recent years. Multiple immune regimes, especially combination therapies, have been proved to be safe and tolerated. Although immune combination strategies may not be effective for all patients, these previous studies enlighten us and encourage us to further explore other combination strategies targeting specific patients and specific tumor backgrounds, in addition to improving present drug dosage forms.

## Figures and Tables

**Table 1:** Current ongoing clinical trials of anti-PD1 and anti-CTLA4 in recurrent glioma.

Study Number	Intervention	Disease	Phase	Status
NCT04145115	Ipilimumab + nivolumab	Recurrent Astrocytoma, IDH-Mutant, Grade 4/Recurrent Glioblastoma, IDH Wildtype/Secondary Glioblastoma	2	Recruiting
NCT04323046	Ipilimumab + nivolumab	Recurrent Glioblastoma/Recurrent Malignant Glioma/Recurrent Grade III Glioma	1	Active, not recruiting
NCT03890952	Nivolumab + bevacizumab (BEV)	Recurrent Adult Brain Tumor	2	Active, not recruiting
NCT05540275	Sintilimab plus Bevacizumab	Recurrent Glioblastoma	2	Not yet recruiting
NCT02658279	Pembrolizumab	Glioma/Recurrent Malignant Glioma	Not applicable	Active, not recruiting
NCT02359565	Pembrolizumab	Recurrent (Refractory) Brain Neoplasm/Recurrent Childhood Ependymoma/Recurrent (Refractory) Diffuse Intrinsic Pontine Glioma/Recurrent (Refractory) Medulloblastoma/Refractory Ependymoma	1	Recruiting
NCT03722342	Pembrolizumab + TTAC-0001	Recurrent Glioblastoma	1	Active, not recruiting
NCT02337686	Pembrolizumab	Recurrent Glioblastoma/Recurrent Gliosarcoma	2	Active, not recruiting
NCT04201873	Pembrolizumab + dendritic cell tumor cell lysate vaccine	Recurrent Glioblastoma	1	Recruiting
NCT04013672	Pembrolizumab + SurVaxM + Sargramostim + Montanide ISA 51	Recurrent Glioblastoma	2	Active, not recruiting
NCT05465954	Pembrolizumab + efineptakin alfa	Recurrent Glioblastoma, IDH-Wildtype/Recurrent Gliosarcoma	2	Recruiting
NCT04479241	Pembrolizumab + lerapolturev	Recurrent Glioblastoma	2	Active, not recruiting
NCT05053880	Pembrolizumab + ACT001	Recurrent Glioblastoma Multiforme (GBM)	1/2	Recruiting
NCT05463848	Pembrolizumab + Olaparib + Temozolomide	Recurrent Glioblastoma	2	Recruiting

**Table 2:** Current ongoing clinical trials of oncolytic virus in recurrent glioma.

Study Number	Intervention	Disease	Phase	Status
NCT03152318	rQNestin 34.5 (HSV-1) + Cyclophosphamide	Malignant Glioma of Brain/Astrocytoma/Malignant Astrocytoma/Oligodendroglioma/Anaplastic Oligodendroglioma of Brain (Diagnosis)/Mixed Oligoastrocytoma/Ependymoma/Ganglioglioma/Pylocytic/Pylomyxoid Astrocytoma	1	Recruiting
NCT04482933	G207 (HSV-1)	High Grade Glioma/Glioblastoma Multiforme/Malignant Glioma of Brain/Anaplastic Astrocytoma of Brain/High-Grade Glioma/Anaplastic Glioma/Giant Cell Glioblastoma	2	Not yet recruiting
NCT02062827	M032 (NSC 733972, HSV-1)	Recurrent Glioblastoma Multiforme/Progressive Glioblastoma Multiforme/Anaplastic Astrocytoma or Gliosarcoma	1	Active, not recruiting
NCT03657576	C134 (HSV-1)	Glioblastoma Multiforme of Brain/Anaplastic Astrocytoma of Brain/Gliosarcoma of Brain	1	Active, not recruiting
NCT03896568	Ad5-DNX-2401 (Adenovirus)	IDH1 wt Allele/Recurrent Anaplastic Astrocytoma/Recurrent Glioblastoma/Recurrent Gliosarcoma/Recurrent Malignant Glioma	1	Recruiting
NCT02457845	G207	Supratentorial Neoplasms, Malignant/Malignant Glioma/Glioblastoma/Anaplastic Astrocytoma/PNET/Cerebral Primitive/Neuroectodermal Tumor/Embryonal Tumor	1	Active, not recruiting
NCT03043391	Polio/Rhinovirus Recombinant (PVSRIPO)	Malignant Glioma/Anaplastic Astrocytoma/Anaplastic Oligoastrocytoma/Anaplastic Oligodendroglioma/Glioblastoma/Gliosarcoma/Atypical Teratoid/Rhabdoid Tumor of Brain/Medulloblastoma/Ependymoma/Pleomorphic Xanthoastrocytoma of Brain/Embryonal Tumor of Brain	1	Active, not recruiting
NCT03911388	G207	Neoplasms, Brain/Glioblastoma Multiforme/Glioblastoma of Cerebellum/Neoplasms/Astrocytoma/Astrocytoma, Cerebellar/Neuroectodermal Tumors/Neuroectodermal Tumors, Primitive/Cerebellar PNET, Childhood/Cerebellar Neoplasms	1	Recruiting

**Table 3:** Current ongoing clinical trials of vaccines and CAR-T cell therapies in recurrent glioma.

Study Number	Intervention	Disease	Phase	Status
NCT04943718	Personalized vaccine	Malignant Glioma/Recurrent Glioma	1	Recruiting
NCT05609994	PEPIDH1M vaccine + vorasidenib	Recurrent IDH1 Mutant Low-Grade Glioma	1	Not yet recruiting
NCT03299309	PEP-CMV	Recurrent Medulloblastoma/Recurrent Brain Tumor, Childhood	1	Recruiting
NCT05096481	PEP-CMV + temozolomide + tetanus diphtheria vaccine	High Grade Glioma/Diffuse Intrinsic Pontine Glioma/Recurrent Medulloblastoma	2	Not yet recruiting
NCT01130077	HLA-A2 restricted glioma antigen peptides vaccine + poly-ICLC	Newly Diagnosed and Recurrent Pediatric Glioma	1	Active, not recruiting
NCT05457959	Dendritic cell tumor peptide vaccine + ipilimumab + nivolumab	Diffuse Hemispheric Glioma, H3 G34-Mutant	1	Not yet recruiting
NCT04888611	GSC-DCV + camrelizumab	Recurrent Glioblastoma	2	Recruiting
NCT03360708	Malignant glioma tumor lysate pulsed autologous dendritic cell vaccine	Giant Cell Glioblastoma/Recurrent Glioblastoma/Recurrent Gliosarcoma	1	Active, not recruiting
NCT04201873	Dendritic cell tumor cell lysate vaccine + pembrolizumab	Recurrent Glioblastoma	1	Recruiting
NCT01814813	HSPPC-96 + bevacizumab	Recurrent Glioblastoma/Recurrent Adult Brain Tumor/Gliosarcoma	2	Active, not recruiting
NCT05540873	YYB-103	Recurrent Malignant Glioma	1	Recruiting
NCT04003649	IL13Rα2-specific Hingeoptimized 4-1BB-co-stimulatory CAR/Truncated CD19-expressing Autologous TN/MEM Cells + ipilimumab + nivolumab	Recurrent Glioblastoma/Refractory Glioblastoma	1	Recruiting
NCT04214392	Chlorotoxin (EQ)-CD28 CD3zeta-CD19texpressing CAR T lymphocytes	Recurrent Glioblastoma/Recurrent Malignant Glioma/Recurrent WHO Grade II/III Glioma	1	Recruiting
NCT04185038	SCRICARB7H3(s); B7H3-specific chimeric antigen receptor (CAR) T cell	Diffuse Intrinsic Pontine Glioma/Diffuse Midline Glioma/Recurrent or Refractory Pediatric CNS Tumors	1	Recruiting
NCT03389230	HER2(EQ)BB#/CD19t+ T cells	Glioblastoma/Malignant Glioma/Recurrent or Refractory Glioma/WHO Grade III Glioma	1	Recruiting
NCT03638167	EGFR806-specific chimeric antigen receptor (CAR) T cell	Recurrent or Refractory Pediatric CNS tumors	1	Active, not recruiting
NCT03500991	HER2-specific chimeric antigen receptor (CAR) T cell	Recurrent or Refractory Pediatric CNS tumors	1	Recruiting
NCT05577091	Inverse correlated dual target, truncated IL7Ra modified CAR-expressing autologous T lymphocytes	Recurrent Glioblastoma	1	Not yet recruiting
NCT04717999	NKG2D CAR-T	Recurrent Glioblastoma	Not applicable	Not yet recruiting
NCT04385173	B7-H3 CAR T + Temozolomide	Recurrent/Refractory Glioblastoma	1	Recruiting
NCT04077866	B7-H3 CAR T + Temozolomide	Recurrent/Refractory Glioblastoma	1/2	Recruiting
NCT02208362	IL13Rα2-specific Hingeoptimized 4-1BB-co-stimulatory CAR/Truncated CD19-expressing Autologous TN/MEM cells/T lymphocytes	Recurrent (Refractory) Glioblastoma/Recurrent (Refractory) Malignant Glioma/Recurrent (Refractory) WHO II/III Glioma	1	Active, not recruiting
